# Electrophysiological Evidence of Stroboscopic Training in Elite Handball Players: Visual Evoked Potentials Study

**DOI:** 10.5114/jhk/169443

**Published:** 2023-10-11

**Authors:** Teresa Zwierko, Wojciech Jedziniak, Jarosław Domaradzki, Michał Zwierko, Marlena Opolska, Wojciech Lubiński

**Affiliations:** 1 Institute of Physical Culture Sciences, Laboratory of Kinesiology, Functional and Structural Human Research Center, University of Szczecin, Szczecin, Poland.; 2 Unit of Biostructure, Faculty of Physical Education and Sport, Wroclaw University of Health and Sport Sciences, Wroclaw, Poland.; 3 Department of Team Sports Games, Wroclaw University of Health and Sport Sciences, Wroclaw, Poland.; 4 Institute of Biology, University of Szczecin, Szczecin, Poland.; 5 II Department of Ophthalmology, Pomeranian Medical University, Szczecin, Poland.

**Keywords:** cortical potentials, vision training, team games, visual pathway

## Abstract

Stroboscopic training enhances perceptual cognition and motor skills; however, neurophysiological mechanisms underlying this adaptation are not fully understood. This study aimed to investigate the effects of a six-week stroboscopic training program on the conductivity of the visual pathway in elite handball players, specifically related to their visual processing of retinal location and viewing conditions. The study included 22 handball players who were randomly assigned to an experimental or a control group. Both groups performed handball-specific in-situ tasks, but only the experimental group underwent stroboscopic training. Participants were assessed three times using visually evoked potential recordings measured by P100 implicit time and amplitude under three viewing conditions (dominant eye, non-dominant eye, and binocular) and two retinal locations (extra-foveal and foveal vision) before and after the six-week training period, and again four weeks later. The results showed a significant TIME vs. GROUP effect of P100 implicit time for the dominant eye in extra-foveal vision (F_2,40_ = 5.20, p = 0.010, ηp^2^ = 0.206), extra-foveal binocular viewing (F_2,40_ = 3.32, p = 0.046, ηp^2^ = 0.142), and dominant eye foveal vision (F_2,40_ = 4.07, p = 0.025, ηp^2^ = 0.169). Stroboscopic training significantly improved early visual processing by reducing the P100 implicit time for the dominant eye and binocular vision, particularly in extra-foveal vision. The improvements were more noticeable in the short compared to the long term.

## Introduction

Stroboscopic training is a technique that uses intermittent visual stimuli during motor tasks. This method places greater demands on the visuomotor system, resulting in improved performance under normal vision conditions. Stroboscopic eyewear, also known as shutter glasses, has become increasingly popular in sport-specific training as it allows athletes to train under specific visual conditions. These glasses alternate between clear and opaque states, reducing the amount of visual information and requiring adjustments in the speed of visual processing. To prevent adaptation effects to specific settings and to account for expected improvements in visual and visuomotor performance over time, the frequency (in hertz) and duty cycle (in percent) settings are often modified during the training period, with task demands increasing as the hutter frequency decreases (Hülsdünker et al., 2021a, 2021b).

Stroboscopic visual training improves visual, perceptual-cognitive, and motor skills (Wilkins and Appelbaum, 2020). Under stroboscopic conditions, the amount of visual information available in the environment is greatly reduced, forcing the individual to extract and process relevant environmental information faster. Furthermore, a recent study showed that stroboscopic vision conditions delay the speed of visual motion perception and processing in the central nervous system and reduce the visuomotor reaction speed (Hülsdünker et al., 2023). As a result, individuals following stroboscopic training improve performance under normal vision conditions.

Increasing evidence indicates that stroboscopic training enhances several perceptual-cognitive and motor skills related to motion sensitivity and anticipatory timing (Appelbaum et al., 2011), coincidence-anticipation performance (Ballester et al., 2017; Krawczyk et al., 2023), visuomotor reaction time (Hülsdünker et al., 2021a; Krawczyk et al., 2023), the ability to track multiple objects (Bennett et al., 2018), catching performance (Wilkins and Gray, 2015), depth jump performance (Kroll et al., 2020; Krawczyk et al., 2023), and postural control (Lee et al., 2022). Additional beneficial impacts were noted during sport-specific testing in ice hockey (Mitroff et al., 2013) and badminton (Hülsdünker et al., 2021a). It has been suggested that these benefits are linked to more efficient processing of visual information (Carroll et al., 2021; Wilkins and Appelbaum, 2020), which was further supported by recent neurophysiological findings of decreased activation latency in the visual motion-sensitive middle temporal (MT) area and improved reaction time (Hülsdünker et al., 2021b). Overall, these findings suggest that stroboscopic training can induce functional changes in the visual system, leading to faster sensory processing and improved performance in various contexts. However, the precise neurophysiological mechanisms underlying this adaptation are not yet fully understood.

Therefore, this study objective was to examine how stroboscopic intervention would impact the conductivity of the visual pathway by assessing the P100 implicit time (latency) and amplitude of visual evoked potentials (VEPs), which are key indicators of visual conductivity status (Walsh et al., 2005). The P100 wave is considered the most reliable and stable component of VEPs and is detectable on all scalp regions that correspond to the occipital cortex, with the highestamplitude typically recorded at the inion along the midline (Baiano and Zeppieri, 2022). The wave is characterized by a positive deflection that occurs approximately 100 ms after the presentation of a visual stimulus generated by activity in the V1 region of the primary visual cortex (Di Russo et al., 2005). As such, the P100 wave reflects early visual processing (Haxby et al., 2002), and this VEP waveform component has been extensively studied and is commonly used in clinical and research settings to assess visual pathway conductivity (Leocani et al., 2018). In a recent study conducted by Poltavski et al. (2021), youth ice hockey players trained using a combination of sports vision training (including stroboscopic intervention), and their progress was monitored with VEP tests. The study reported a significant reduction in P100 latency and amplitude following ten weeks of visual training.

Gaining a more comprehensive understanding of stroboscopic training in visual processing requires analysis of retinal location and viewing conditions. The processing of foveal and extra-foveal (peripheral) vision differs in many aspects. Indeed, cone photoreceptor density peaks in the fovea and declines toward the periphery, with more receptors converging on a single peripheral retinal ganglion cell than in the fovea (Curcio et al., 1990). Therefore, foveal vision is characterized by high acuity and contrast sensitivity, whereas peripheral vision allows the perception of a large part of the visual field with decreased acuity and greater uncertainty over the localization of objects (Strasburger et al., 2011). Nonetheless, peripheral and foveal signals are integrated across saccadic eye movements under normal vision conditions to achieve an average perception of the environment (Stewart et al., 2020). While it is clear that foveal and extra-foveal processing are closely connected, their functioning during stroboscopic vision and which system will be more affected by systematic stroboscopic stimulation remains unknown.

The clinical significance of ocular dominance is widely acknowledged, with numerous studies confirming the existence of physiological, sensory, and motor foundations associated with ocular dominance in individuals with normal binocularity and equal refractive errors (Handa et al., 2004; Laby and Kirschen, 2011). Experimental studies have demonstrated better accommodative function (Momeni-Moghaddam et al., 2014), shorter response latency of visual evoked potentials (Nguyen et al., 2019), as well as faster reaction time and accuracy rates in visuomotor tasks (Liu et al., 2021) when participants used their dominant eyes. Hence, it seems likely that a structural (Choi et al., 2016) and functional asymmetry (Liu et al., 2021) between the dominant and the non-dominant eye will influence the variation of VEP components after a stroboscopic intervention.

This study aimed to investigate the effects of a six-week stroboscopic training program on the conductivity of the visual pathway in elite handball players, including their visual processing related to retinal location and viewing conditions. Handball is an open-skill sport that requires superior visual and visuomotor skills to dynamically interact with its ever-changing environment, including maintaining peripheral vision, oculomotor functioning, and visuomotor processing (Blecharz et al., 2022; Hodges et al., 2021; Zwierko et al., 2008). The training program involved specific handball exercises performed with and without stroboscopic eyewear. Based on the results of previous studies (Hülsdünker et al., 2021a, 2021b; Poltavski et al., 2021), it was hypothesized that the use of stroboscopic eyewear during the training program would enhance visual processing speed compared to the same exercises performed without the eyewear.

## Methods

### Participants

According to Poltavski et al. (2021), the multivariate effect of ten weeks of visual training on P100 latency and amplitude had a partial eta square (ηp^2^) of 0.13 and 0.46, respectively. Based on these findings, the effect size for the current study was calculated as 0.38. Power analysis using a *p*-value of 0.05 and a test power of 0.95 indicated a required sample size of 20 participants (Faul et al., 2007). The study initially included 24 participants randomly assigned to either the stroboscopic or the control group, with 12 participants each. However, due to injuries to two players, the study included 11 participants in each group. The study sample encompassed 22 highly experienced male handball players, including 20 right-handed and two left- handed, with a mean age of 24.59 years (± 5.4). The participant’s training experience equated to 12.95 years (± 3.3), with a weekly training load of 13.29 (± 4.1) hours. Handball players were classified as elite players based on their participation in the highest league of handball competition (Superliga) for the past four seasons. Before the experiment, all participants received information about the study protocol and provided written consent. The local bioethical committee (No. 11/KB/V/2017) provided study approval, and the study conformed with the principles of the Declaration of Helsinki. Raw data for this study are available in the repository at https://repod.icm.edu.pl/dataset.xhtml?persistentId=doi:10.18150/0SVGKH. Table 1 provides a comparative analysis of the characteristics of the experimental and control groups.

**Table 1:** Descriptive (mean ± standard deviation) characteristics of the groups.

	Stroboscopic group (n =11)	Control group (n = 11)	*p*
Age (years)	23.7 ± 5.7	25.4 ± 5.7	0.485
Body height (cm)	186.1 ± 7.0	188.0 ± 6.3	0.512
Body mass (kg)	86.8 ± 13.1	90.9 ± 15.9	0.519
Sports experience (years)	12.7 ± 3.6	13.1 ± 3.2	0.761
The effective time duration of training intervention (min/week)	59.2 ± 1.2	58.9 ± 1.4	0.543

Note: p-values correspond to the t-test

### Measures

#### Visual Evoked Potentials

**Figure 1:**
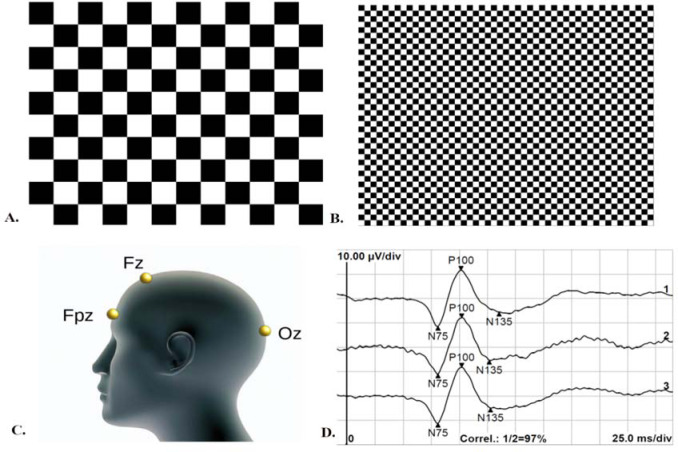
Visual evoked potentials recording protocol; (A) Stimulus pattern for processing extra-foveal retinal location: black and white reversing checkerboard with check sizes of 1°4'; (B) Stimulus pattern for processing foveal retinal location: black and white reversing checkerboard with check sizes of 0°16'; (C) ISCEV electrode placement: active electrode at the Oz point of the occipital region, reference electrode at the Fz point, and ground electrode at the Fpz point; (D) Example of visual evoked potentials recordings of foveal retinal processing under binocular viewing: two subsequence measurements (labeled 1 and 2) and the average value of the two recordings (labeled 3). The recordings are marked with the N75, P100, and N135 waves.

This study followed the International Society for Clinical Electrophysiology of Vision (ISCEV) protocol (Odom et al., 2016) to ensure standardized and consistent recording of the VEPs. For VEP recordings, monocular and binocular stimulation with central fixation was utilized without pupil dilation, while appropriate refraction correction was applied at a distance of one meter. The recording electrodes consisted of an active gold disk electrode placed over the visual cortex at Oz, a reference gold disc electrode at Fz, and a ground gold disc electrode at Fpz on the forehead (Figure 1, panel C), with the acceptable electrode impedance set at <5 kΩ. Stimulus variables consisted of a black and white reversing checkerboard with two check sizes, measuring 0°16' and 1°4', respectively (Figure 1, panels A and B). The white elements had a luminance of 120 cd/m2, with a 97% contrast between black and white squares. The recording system used an amplifier range of ±100 μV/div, 1–100 Hz filters, a sweep time of 300 ms, and an artifact rejection threshold of 95%. Two trials of 100 artifact-free sweeps for each check size were obtained and then averaged offline. The analysis included the amplitude and peak time of the P100 wave (Figure 1, panel D). The viewing conditions (monocular dominant eye, monocular non-dominant eye, and binocular) were randomized during VEP recording. The hole-in-card test (Walls, 1951) determined ocular dominance, and the procedure involved participants holding a 30-cm square rigid board with 8-inch sides and a 3-cm hole in the center. The board was then placed in the lap and raised with arms fully extended while fixating on a small (2 cm) circular target on a computer screen positioned 2.5 m away. The experimenter then used alternate occlusion to determine which eye was fixating on the target. This process was repeated three times, with arms fully extended, partially flexed, and fully flexed, with the board just a few centimeters from the nose. The entire procedure was repeated, with the participant lowering the board from above the target.

To minimize potential bias and ensure that the observed effects were truly due to the stroboscopic protocol, participants were divided into two groups: a stroboscopic group and a non-stroboscopic group. Each group was tested on a separate day under the same conditions, with testing sessions starting at 9 am. Athletes had no special dietary requirements during the clinical measurements. To avoid any interference from physical fatigue (Zwierko et al., 2010a), VEP recordings were conducted on a day off from training. Participants were familiarized with the clinical tests.

### Design and Procedures

#### Training Intervention

The study consisted of a six-week training program followed by a four-week retention interval and was conducted during the regular season. Additionally, the test day maintained the same conditions for all measurements (pre-test, post-test, and retention test). During the six-week training period, the experimental and control groups underwent an equal training protocol three times a week under either stroboscopic or normal vision conditions. The duration of the intervention was similar to previous research protocols (Hülsdünker et al., 2021a, 2021b).

**Figure 2:**
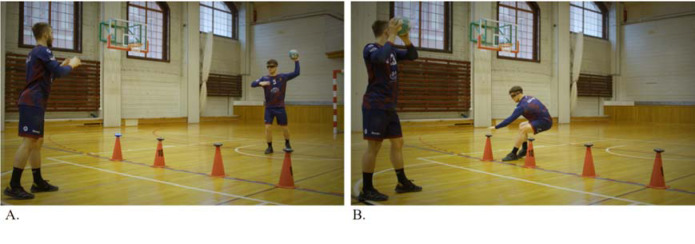
A graphical illustration of a study protocol example. (A) The player stands with their back towards the 6-m line and passes the ball to their partner. Four light discs are set up on the 9-m line, which randomly activates in either red or blue. (B) After the pass, light signals appear (blue or red). The player runs to the lights and deactivates the designated color by touching the disc (red color with their left hand and blue color with their right hand) as fast as possible, then returns to their position and is ready for the next pass.

The training program comprised three different protocols. Protocol A involved five “Ball catch drills” using external light stimuli (as shown in Figure 2), different ball sizes (such as a tennis ball), and varying colors (blue and red). Protocol B included five “Partner passing drills” in the frontal position, including visual search, time pressure, and second ball exercises. Protocol C involved four “Handball-specific passing drills” in the frontal and side positions, which used varying distances between partners and changes in running direction.

Stroboscopic eyewear (Senaptec Strobe, OR, USA) provided the stroboscopic vision conditions. During the intervention period, the frequency (Hz) and duty cycle (%) settings of the glasses were modulated to avoid the effects of adaptation to specific settings and to obtain the expected improvement in visual-motor skills. Six different settings of the gleam frequency and duty cycle were defined, with increasing task difficulty and training duration according to the methodology adopted by Hülsdünker et al. (2021a). Specific settings for each training period were week 1 (5 Hz, 50%), week 2 (13 Hz, 50%), week 3 (11 Hz, 50%), week 4 (10 Hz, 50%), week 5 (9 Hz, 60%), and week 6 (9 Hz, 70%).

### Statistical Analysis

All analyses utilized JASP statistical software version 16.1. Descriptive statistics are presented as means and standard deviations. The Shapiro-Wilk test assessed data normality, and the Levene’s test confirmed the homogeneity of variance (*p*> 0.05). A mixed-model analysis of variance (ANOVA) assessed each viewing condition and retinal location for the intra-subject factor TIME (pre, post, and retention) and the inter-subject factor GROUP (stroboscopic and control). The Mauchley’s test revealed no sphericity breach from examination of the repeated measures factor (*p*> 0.05). Post hoc comparisons employed the Holm-Bonferroni procedure, with statistical significance set at *p*< 0.05. Effect sizes were reported using Cohen’s *d* and partial eta ηp^2^ for *t*-tests and F-tests, respectively. Cohen’s criteria (1988) were used to interpret the effect sizes, where values of 0.2, 0.5, and 0.8 represented small, medium, and large effect sizes, respectively, and values of 0.01, 0.06, and 0.14 represented small, medium, and large effect sizes, respectively, for partial eta squared (ηp^2^).

## Results

Control analyses revealed no differences between the groups in anthropometric measurements, mean weekly training time or training exposure time (Table 1). Table 2 displays the descriptive statistics for the sample in the pre- test, post-test, and retention conditions for the stroboscopic and control groups.

**Table 2:** Descriptive statistics of visually evoked potential variables, including P100 implicit time (ms) and P100 amplitude (μV) (below), in the stroboscopic and control groups during pre-tests, post-tests, and retention tests.

Group	*Extra-foveal vision*			*Foveal vision*		
	pre	post	retention	pre	post	retention
*Non-dominant eye*						
stroboscopic	106.5 ± 3.3	106.7 ± 3.2	107.0 ± 3.9	108.8 ± 3.7	108.3 ± 3.1	109.4 ± 3.6
	16.1 ± 4.0	14.8 ± 3.6	14.2 ± 2.9	16.9 ± 4.4	16.7 ± 3.5	16.8 ± 4.0
control	107.5 ± 2.9	107.6 ± 3.0	108.0 ± 2.8	109.7 ± 3.9	109.5 ± 4.2	109.9 ± 4.2
	16.6 ± 3.1	16.7 ± 4.0	16.8 ± 4.7	16.8 ± 5.4	17.4 ± 4.8	17.6 ± 5.5
*Dominant eye*						
stroboscopic	105.6 ± 3.4	103.9 ± 2.6	105.1 ± 2.7	108.6 ± 3.3	107.2 ± 3.9	109.3 ± 3.0
	16.2 ± 3.6	15.7 ± 2.8	15.8 ± 2.4	18.1 ± 4.3	16.15 ± 3.4	16.1 ± 4.2
control	105.6 ± 2.7	105.9 ± 3.2	106.3 ± 2.4	108.6 ± 2.9	108.7 ± 3.1	108.5 ± 3.0
	16.1 ± 3.7	16.0 ± 3.8	15.5 ± 3.1	17.7 ± 4.7	16.7 ± 4.1	17.2 ± 4.6
*Binocular*						
stroboscopic	103.7 ± 3.6	102.8 ± 3.3	104.2 ± 3.1	105.8 ± 3.6	105.3 ± 3.1	106.5 ± 2.9
	16.9 ± 3.6	15.4 ± 4.2	15.9 ± 4.1	18.9 ± 4.9	16.9 ± 3.6	17.5 ± 3.4
control	104.2 ± 2.5	104.7 ± 2.6	105.1 ± 2.3	106.7 ± 2.9	107.1 ± 3.0	107.0 ± 3.4
	17.1 ± 4.4	16.4 ± 4.4	16.7 ± 3.7	19.1 ± 5.7	18.9 ± 4.0	19.4 ± 5.1

### Extra-Foveal Vision

#### Non-Dominant Eye

The results indicate no significant effects on the P100 implicit time and amplitude of the non-dominant eye across TIME, GROUP, and TIME x GROUP conditions. Specifically, there were no significant effects of TIME (F_2,40_ = 1.18, *p* = 0.317, ηp^2^ = 0.056) or GROUP (F_1,20_ = 0.56, *p* = 0.462, ηp^2^ = 0.027), nor were there any significant interaction effects for the P100 implicit time (F_2,40_ = 0.03, *p* = 0.972, ηp^2^ = 0.001). Similarly, for the P100 amplitude, there were no significant effects of TIME (F_2,40_ = 1.55, *p* = 0.224, ηp^2^ = 0.072) or GROUP (F_1,20_ = 1.26, *p* = 0.275, ηp^2^ = 0.059), nor were there any significant interaction effects for the P100 amplitude (F_2,40_ = 2.64, *p* = 0.084, ηp^2^ = 0.12).

#### Dominant Eye

**Figure 3:**
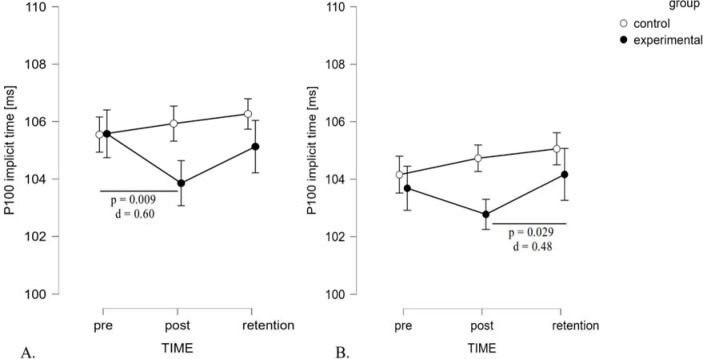
Interaction plot of TIME x GROUP effects on extra-foveal vision, shown for the dominant eye (panel A) and binocular viewing (panel B).

Regarding the dominant eye in extra-foveal vision, the findings revealed a significant effect of TIME (F_2,40_ = 81.54, *p* = 0.040, ηp^2^ = 0.15) and a significant interaction of TIME x GROUP (F_2_,_40_ = 5.20, *p* = 0.010, ηp^2^ = 0.21) (Figure 3, panel A). However, the between-subject effect of GROUP was non-significant (F_1,20_ = 0.84, *p* = 0.371, ηp^2^ = 0.04). The significant interaction suggests that changes between tests (pre-post-retention) were quite different in both groups. Post hoc analysis revealed that the stroboscopic group exhibited a significant reduction of implicit time in post-test scores compared to baseline values, with a medium effect size (105.57 ± 3.37 ms vs. 103.86 ± 2.63 ms, *p* = 0.009, *d* = 0.60). For the P100 amplitude, there were no significant effects of TIME (F_2,40_ = 2.62, *p* = 0.085, ηp^2^ = 0.12) or GROUP (F_1,20_ = 0.02, *p* = 0.892, ηp^2^< 0.01), nor any significant interaction effect for the P100 amplitude (F_2,40_ = 1.10, *p* = 0.344, ηp^2^ = 0.05).

#### Binocular

ANOVA of the binocular viewing P100 implicit time revealed a significant effect for TIME (F_2,40_ = 4.72, *p* = 0.014, ηp^2^ = 0.19) and no effect for GROUP (F_1,20_= 0.85, *p* = 0.37, ηp^2^ = 0.04). Furthermore, an interaction was observed between TIME and GROUP factors (F_2,40_ = 3.32, *p* = 0.046, ηp^2^ = 0.14). Similarly to the dominant eye results, changes between tests were related to the group. Only in the stroboscopic group did post hoc tests show significant differences between the post-test and the retention test (102.77±3.29vs. 104.16±3.08ms, *p* = 0.029, *d* = 0.48) (Figure 3, panel B). The P100 amplitude showed a significant effect for TIME (F_2,40_ = 4.611, *p* = 0.016, ηp^2^ = 0.19), no effect for GROUP (F_1,20_= 1.15, *p* = 0.706, ηp^2^ = 0.01), nor any significant interaction effect (F_2,40_ = 0.721, *p* = 0.492, ηp^2^ = 0.04).

### Foveal Vision

#### Non-Dominant Eye

There were no significant effects of TIME (F_2,40_ = 0.24, *p* = 0.789, ηp^2^ = 0.01) or GROUP (F_1,20_ = 0.30, *p* = 0.589, ηp^2^ = 0.02), nor were there any significant interaction effects for the P100 implicit time (F_2,40_ = 0.36, *p* = 0.703, ηp^2^ = 0.02). Similarly, for the P100 amplitude, there were no significant effects of TIME (F_2,40_ = 1.55, *p* = 0.224, ηp^2^ = 0.07) or GROUP (F_1,20_ = 0.05, *p* = 0.820, ηp^2^ = 0.003), nor were there any significant interaction effects for the P100 amplitude (F_2,40_ = 0.64, *p* = 0.530, ηp^2^ = 0.03).

#### Dominant Eye

The analysis of the dominant eye did not reveal a significant effect of TIME (F_2,40_ = 2.99, *p* = 0.062, ηp^2^ = 0.13). However, the interaction of TIME x GROUP was significant (F_2,40_ = 4.07, *p* = 0.025, ηp^2^ = 0.17), whereas the GROUP factor was not significant (F_1,20_ = 0.04, *p* = 0.840, ηp^2^ = 0.002). Post hoc tests in the stroboscopic group demonstrated significant differences between the post-test and retention test (107.2 ± 3.9 ms vs. 109.3 ± 3.0 ms, *p* = 0.01, *d* = 0.66). For the P100 amplitude, there was a significant effect of TIME (F_2,40_ = 5.61, *p* = 0.007, ηp^2^ = 0.22) and no significant effect of GROUP (F_1,20_ = 0.01, *p* = 0.802, ηp^2^ = 0.01). The interaction of TIME x GROUP was also non-significant (F_2,40_ = 1.23, *p* = 0.304, ηp^2^ = 0.06).

#### Binocular

The analyses of P100 implicit time did not reveal a significant effect for either TIME (F_2,40_ = 1.47, *p* = 0.242, ηp^2^ = 0.068) or GROUP (F_1,20_ = 0.64, *p* = 0.434, ηp^2^ = 0.03) factors. *The interaction between factors did not* exert significant effects (F_2,40_ = 2.37, *p* = 0.189, ηp^2^ = 0.08). Similarly, for the P100 amplitude, there were no significant effects of TIME (F_2,40_ = 2.20, *p* = 0.124, ηp^2^ = 0.10) or GROUP (F_1,20_ = 0.54, *p* = 0.472, ηp^2^ = 0.03), nor were there any significant interaction effects (F_2,40_ = 2.06, *p* = 0.141, ηp^2^ = 0.09).

## Discussion

The current study examined the influence of a 6-week stroboscopic training program on the P100 implicit time and amplitude in both the dominant and the non-dominant eye, in binocular vision, and during foveal and extra-foveal viewing. The results revealed that stroboscopic training decreased the P100 implicit time for the dominant eye, particularly in extra-foveal vision, and exerted a minor effect on the P100 amplitude. Nevertheless, no significant variations were observed in the P100 implicit time and amplitude of the non-dominant eye across all analyzed conditions. Additionally, the stroboscopic intervention impacted extra-foveal vision favorably under binocular viewing conditions. Furthermore, no long-term effects were detected for VEP variables when assessed four weeks after the conclusion of the experiment.

This study provides evidence that stroboscopic training can enhance visual processing at the sensory level by accelerating the signal conductivity in the visual pathway. Specifically, we observed faster V1 region activation of the primary visual cortex when the dominant eye and binocular vision were stimulated in extra-foveal vision. This finding is consistent with the research conducted by Poltavski et al. (2021), who observed improvements in visual signal processing speed in young ice hockey players after ten weeks of sports vision training that included stroboscopic vision. Specifically, they found a reduction in the time between retinal stimulation and excitation of neurons in the primary visual cortex, as measured by the P100 wave of VEPs during binocular conditions in response to checkerboard stimuli with 2° checks presented at 85% contrast and a temporal frequency of 1 Hz. The current study focused on binocular viewing in an extra-foveal location, using stimuli with 1°4' checks presented at 97% contrast and a temporal frequency of 1 Hz. Similarly, a significant improvement in visual processing was revealed, as indicated by a reduction in P100 implicit time during binocular conditions, but only for larger extra-foveal stimuli, not for smaller foveal stimuli (small pattern size of 0°16' checks). Multiple studies have found that athletes engaged in fast-paced sports like volleyball, tennis, squash, fencing, and karate exhibit faster P100 implicit time in response to checkerboard stimuli when compared to non-athletes (Del Percio et al., 2007; Taddei et al., 1991; Zwierko et al., 2010b).

The novelty of the study findings is related to the observation that stroboscopic training has more impact on visual processing improvement in the dominant eye over the non-dominant eye, which is supported by the decrease in the P100 implicit time for the dominant eye. Previous research has shown that the dominant eye has functional advantages, including better feature and conjunction search performance (Shneor and Hochstein, 2006, 2008) and improved performance in visuomotor tasks (Liu et al., 2021). Furthermore, a study by Hofeldt et al. (1996) found that reducing the contrast with a neutral density filter placed over the dominant eye had a greater negative impact on binocular motion task performance (baseball hitting) than when placed over the non-dominant eye. The current study findings suggest that visual disturbances caused by stroboscopic light during exercise indicate that perceptual processing priority is a function of the dominant eye. This finding aligns with prior research that demonstrated the superior performance of the dominant eye during binocular viewing conditions (Foutch and Bassi, 2020; Handa et al., 2004; Hofeldt et al., 1996). In future research, it would be valuable to design an experiment examining the effects of a stroboscopic intervention that primarily stimulates the non-dominant eye.

It is widely accepted that there are two distinct visual pathways, the magnocellular and parvocellular pathways, that process different types of visual information. The magnocellular stream is responsible for processing information on the spatial location and motion of visual stimuli, while the parvocellular is responsible for processing the color and form of visual stimuli. The magnocellular pathway is sensitive to low-contrast, low-spatial frequency, and achromatic stimuli, making it crucial for detecting where things are. Meanwhile, the parvocellular pathway is sensitive to chromatic and stationary stimuli and high-spatial frequencies, making it essential for detecting what things are. The training intervention included stimulation of both pathways, with a large effect on extra-foveal vision and the dominant eye. It is thought that parvocellular-biased objects seen through the dominant eye are somehow more discernible than those seen by the non-dominant eye (Foutch and Bassi, 2020), which may partially explain the findings observed in the study. The chessboard pattern stimulation and achromatic high-contrast stimulation ISCEV VEPs used in this study mainly concern the parvocellular pathway (Benedek et al., 2016). Both the magnocellular and parvocellular streams pass through the lateral geniculate bodies and make synaptic connections with different layers of the primary visual cortex, thus are related to the dorsal and ventral streams. There is evidence of mutual interaction between the dorsal and ventral pathways, thus stroboscopic stimulation of the dorsal pathway can also affect the ventral pathway (van Polanen and Davare, 2015). To further enhance our understanding, it is imperative to prioritize the evaluation of stroboscopic training’s impact on the function of the magnocellular pathway. The potentials of this pathway may effectively distinguish between the two parallel visual systems (Tobimatsu et al., 1995).

A study by Poltavski et al. (2021) found that athletes who underwent vision training for ten weeks exhibited a reduction in the P100 amplitude compared to their baseline. Those authors suggested that this decrease may be due to the inhibition of irrelevant visual information. However, the current study only found a decreased P100 amplitude during extra-foveal vision under binocular viewing conditions and foveal vision under monocular viewing conditions of the dominant eye. As such, it is difficult to confirm whether these findings validate Poltavski’s previous observations, although there was a tendency toward this effect in the current study.

The current findings offer new perspectives on the neurophysiological mechanisms of stroboscopic intervention on visual system adaptation. Nonetheless, it is important to acknowledge the limitations of this study. First, the sample size calculation was based on a previous work that also used stroboscopic intervention in vision training. However, the sample size may not have been sufficient to determine significant groupeffects in the current study. Therefore, future studies should consider including more participants to ensure greater statistical power. Prior research has indicated that gender is an important variable affecting the VEP (Sharma et al., 2015), but this study only included male adults. As such, future studies on males and females will allow for the generalizability of results to other populations. Gaining a more thorough understanding of visual system adaptations following stroboscopic training by assessing VEPs that activate the magnocellular visual pathway is required to complement existing research. Indeed, a study by Modjtahedi et al. (2014) investigating differences in magnocellular function between athletes and non-athletes revealed that sports participation influences visual cortical plasticity. Finally, our research does not provide an answer to the question of how the changes observed in visual processing can impact athletes' on-field performance. It is crucial for sports practice to uncover the effects of vision training on athletes' sport-specific movements (far transfer) (Kohmura et al., 2019; Witkowski et al., 2021), which in our case requires additional analysis.

In conclusion, the findings of this study indicate that a six-week stroboscopic training program has a significant effect on early visual processing by decreasing the P100 implicit time for the dominant eye and binocular vision, particularly in the extra-foveal vision, with more significant improvements observed in the short compared to the long term.
